# Predictive value of cell population data with Sysmex XN-series hematology analyzer for culture-proven bacteremia

**DOI:** 10.3389/fmed.2023.1156889

**Published:** 2023-06-01

**Authors:** Yuki Miyajima, Hideki Niimi, Tomohiro Ueno, Atsushi Matsui, Yoshitsugu Higashi, Nozomi Kojima, Mari Kono, Yosuke Iwasaki, Kentaro Nagaoka, Yoshihiro Yamamoto, Isao Kitajima

**Affiliations:** ^1^Department of Clinical Infectious Diseases, Toyama University Hospital, Toyama, Japan; ^2^Department of Clinical Laboratory and Molecular Pathology, Toyama University Hospital, Toyama, Japan; ^3^First Department of Internal Medicine, Toyama University Hospital, Toyama, Japan; ^4^Gene Technology Group, Reagent Engineering, Sysmex Corporation, Hyogo, Japan; ^5^R&D Center Asia Pacific, Sysmex Asia Pacific Pte Ltd, Singapore, Singapore; ^6^Scientific Research, Scientific Affairs, Sysmex Corporation, Hyogo, Japan

**Keywords:** cell population data, bacteremia, sepsis, XN-series, procalcitonin, presepsin, interleukin-6

## Abstract

**Background:**

Cell population data (CPD) parameters related to neutrophils, such as fluorescent light intensity (NE-SFL) and fluorescent light distribution width index (NE-WY), have emerged as potential biomarkers for sepsis. However, the diagnostic implication in acute bacterial infection remains unclear. This study assessed the diagnostic value of NE-WY and NE-SFL for bacteremia in patients with acute bacterial infections, and those associations with other sepsis biomarkers.

**Methods:**

Patients with acute bacterial infections were enrolled in this prospective observational cohort study. For all patients, a blood sample, with at least two sets of blood cultures, were collected at the onset of infection. Microbiological evaluation included examination of the blood bacterial load using PCR. CPD was assessed using Automated Hematology analyzer Sysmex series XN-2000. Serum levels of procalcitonin (PCT), interleukin-6 (IL-6), presepsin, and CRP were also assessed.

**Results:**

Of 93 patients with acute bacterial infection, 24 developed culture-proven bacteremia and 69 did not. NE-SFL and NE-WY were significantly higher in patients with bacteremia than in those without bacteremia (*p* < 0.005, respectively), and were significantly correlated with the bacterial load determined by PCR (*r* = 0.384 and *r* = 0.374, *p* < 0.005, respectively). To assess the diagnostic value for bacteremia, receiver operating characteristic curve analysis was used. NE-SFL and NE-WY showed an area under the curve of 0.685 and 0.708, respectively, while those of PCT, IL-6, presepsin, and CRP were 0.744, 0.778, 0.685, and 0.528, respectively. Correlation analysis showed that the levels of NE-WY and NE-SFL were strongly correlated with PCT and IL-6 levels.

**Conclusion:**

This study demonstrated that NE-WY and NE-SFL could predict bacteremia in a manner that may be different from that of other indicators. These findings suggest there are potential benefits of NE-WY/NE-SFL in predicting severe bacterial infections.

## Introduction

Sepsis is a life-threatening organ dysfunction due to a dysregulated host response to infection. It is a major problem in hospitalized patients worldwide, with high mortality (1). To prevent the progression of sepsis into septic shock or multiple organ failure, early and rapid diagnosis and management are crucial (2). Positive blood culture is still considered the gold standard for diagnosis and detection of bacterial sepsis. However, blood culture has several disadvantages, including long turnaround times and low sensitivity (3, 4). Recently, several serum (or plasma) biomarkers have been proposed for the timely diagnosis and prognostications of patients with sepsis, including C-reactive protein (CRP), procalcitonin (PCT), presepsin, and interleukin 6 (IL-6). However, these sepsis biomarkers also have several disadvantages for routine assessment in septic patients, such as insufficient diagnostic performance with lower specificity, inability to detect causative pathogens, and high costs (3, 4).

Cell population data (CPD) obtained using hematology analyzers has recently attracted attention as a new method for early detection of sepsis, which has enabled the expansion of information available from the complete blood count (5). The Sysmex hematology analyzers such as XN-series can detect the activation of neutrophils, lymphocytes, and monocytes in real time, and in an accurate and reproducible manner. It is based on fluorescent-flow cytometry, using blood-cell membrane surfactant reagents, and fluorescence dyes specific for staining nucleic acids and proteins (6, 7).

Among CPD generated by Sysmex XN analyzers, fluorescent light intensity of the neutrophil area (NE-SFL) and/or fluorescent light distribution width index of the neutrophil area (NE-WY) have been reported as potential biomarkers for sepsis or bacteremia. A few studies have investigated the diagnostic utility of NE-SFL and NE-WY for predicting bacteremia (8–11). Park et al. (8) reported that NE-SFL and NE-WY showed high AUC of 0.909 and 0.905, respectively, for the detection of culture-proven sepsis in their study cohort, which consisted of 130 sepsis patients and 280 normal controls. Lemkus et al. (11) reported that NE-SFL and NE-WY showed high AUC of 0.84 and 0.78 for the detection of culture-proven bacteremia in their study cohort, which consisted of 23 patients with bacteremia and 13 healthy controls. These studies demonstrated the high predictive potential of NE-SFL/NE-WY for detection of bacteremia, when compared with the healthy control. However, it remains unclear whether NE-SFL/NE-WY could predict the presence of bacteremia among patients with acute bacterial infections.

In the present study, we evaluated the accuracy and usefulness of CPD, NE-SFL and NE-WY, as biomarkers for culture-proven bacteremia in hospitalized patients, in comparison with the other commercialized sepsis biomarkers in Japan, including CRP, PCT, presepsin, and IL-6. Furthermore, we measured the bacterial load in the blood using polymerase chain reaction (PCR) to determine whether NE-SFL/NE-WY is truly affected by the presence of bacteria in the blood.

The primary objectives of this study were to assess the diagnostic value and clinical utility of NE-SFL/NE-WY for bacteremia in hospitalized patients who developed acute infection. The secondary objective was to assess the association between NE-SFL/NE-WY and commercialized sepsis biomarkers.

## Methods

### Study design

This prospective observational study was primarily designed to investigate the clinical utility of CPD obtained using Automated Hematology analyzer Sysmex series XN-2000 (Sysmex XN-2000; Sysmex Corporation, Japan) and was approved by the Ethics Committee of Toyama University Hospital (Approval No.29–152) in accordance with the tenets of the Helsinki Declaration. We recruited all consecutive patients who developed acute infections at the Toyama University Hospital between July 1, 2017, and January 31, 2018. Informed consent was obtained from all the patients.

### Study participants and protocol

The inclusion criteria were as follows: (1) men or women aged ≥18 years (2) patients who developed acute infection, clinically diagnosed as bacterial in origin, and (3) culture examinations (at least two sets of blood cultures) submitted before antimicrobial therapy.

Isolation of bacteria from at least one set of blood culture was defined as confirmed bacteremia (culture-proven bacteremia). If less virulent bacterial species, such as coagulase-negative staphylococci, *Bacillus*, *Corynebacterium*, or *Propionibacterium*, were identified after 48–72 h of incubation with only one bottle or one set of bottles, it was diagnosed as contamination, as described in the previous report (12, 13).

According to the Third International Consensus Definition for Sepsis and Septic Shock (Sepsis-3) (14), ‘definite sepsis’ was defined as an increase in Sequential Organ Failure Assessment (SOFA) score ≥ 2 at the onset of infection.

**Figure 1:**
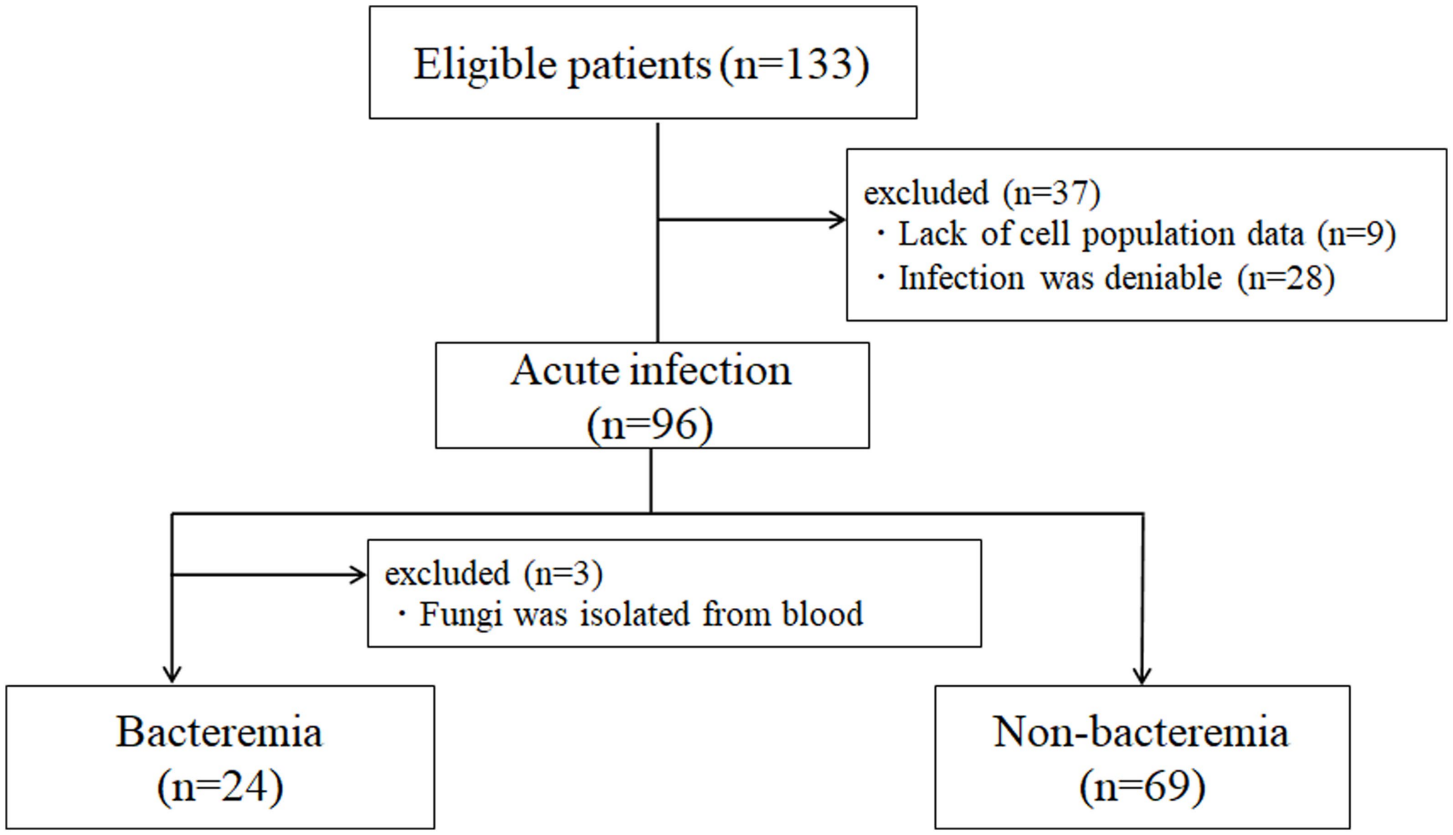
Flow chart showing patient selection.

The exclusion criteria were as follows: (1) deniable acute infection, that is, acute exacerbation of collagen disease, tumor fever and (2) lack of sufficient clinical information, and (3) definite diagnosis of fungemia. The details of patient enrollment are shown in Figure 1.

### Sample collection

Blood samples were collected from each participant at the onset of infection. We obtained data on selected CPD of neutrophils from the blood sample database of our central medical laboratory.

Control blood samples were obtained from healthy immunocompetent volunteers (*n* = 37) at Toyama University Hospital. The volunteers were hospital staff with no known underlying disease. Blood sampling was conducted under afebrile conditions, and serum was stored and utilized for CPD. Written informed consent was obtained from all the volunteers prior to the blood sampling.

### Cell population data

CPD were obtained with Sysmex XN-2000 (Sysmex Corporation, Kobe, Japan), as previously described (8). Briefly, the leukocyte differential channel discriminates leukocytes, and the signals are plotted in a scattergram (WDF). The signals obtained from the three-axes after preincubation with unique surfactant reagents and fluorescence staining are analyzed and calculated according to the distribution width. The optical signals along the X-axis (side scatter) are proportional to the internal complexity; fluorescence along the Y-axis correlates with the nucleic acid content, while forward scatter (Z-axis) is related to cell size (5). The morphological and functional characteristics of the whole CPD panel measured using Sysmex XN-2000 are summarized in Supplementary Table S1.

### Sepsis biomarkers

The serum levels of CRP, PCT, IL-6, and presepsin were measured as sepsis biomarkers, at the time of enrollment. Among the biomarkers, IL-6 and presepsin were measured by SRL, Inc. and LSI Medience Corporation, while the others were measured in the laboratory at our hospital, as routine examinations (using commercialized test reagents and automated analysis biochemistry system, Cobas ^®^ 8,000; Roche Diagnostics K.K., Japan).

### Quantitative PCR assay measuring bacterial load in blood

We measured bacterial load in the blood using a PCR assay, as described previously (15). Briefly, DNA was extracted from blood samples using a DNA extraction kit (QIAamp UCP Pathogen Mini Kit; Qiagen, Germany) according to the manufacturer’s instructions. Bacterial universal primers designed to amplify the seven regions of the bacterial 16S ribosomal RNA gene (16S rDNA) (15) were used. Two step of amplification reactions were performed, and the threshold cycle values of amplification in the second PCR were analyzed using the Rotor-Gene Q software program. If no amplification was observed by the 35th cycle in secondary PCR, the sample was defined as containing no bacteria.

### Statistical analysis

Background factors are expressed as median (interquartile range) or number (percentage). To evaluate the differences between the two groups, the Mann–Whitney test and Pearson’s chi-squared test were used to compare continuous and nominal variables, respectively. Receiver operating characteristic (ROC) curves and the respective areas under the ROC curve (AUC) were generated using GraphPad Prism 9 software (GraphPad Software, San Diego, CA, United States). In each ROC analysis, the cutoff value for the detection of bacteremia was determined using the nearest point relative to the left corner of each ROC curve. The association between each pair of sepsis biomarkers or bacterial load was determined using Spearman’s rho correlation coefficient. Statistical significance was set at *p* < 0.05. JMP Pro 16 ((SAS Institute, Cary, NC, United States)) and GraphPad Prism 9 (GraphPad Software) were used for statistical analysis.

## Results

### Study participants

Detailed information on patient enrollment is presented in Figure 1. Among the patients with bacteremia, three were excluded from further analysis as they were confirmed to have fungemia (two for Candida and one for Cryptococcus). One case in which *Bacillus cereus* was isolated from one of the sets of blood culture, after more than 48 h-incubation, was determined as contamination, and subsequently categorized as ‘infection without bacteremia’. Finally, 24 patients with culture-proven bacteremia and 69 patients without bacteremia were included for further analysis.

The clinical characteristics and laboratory findings of the 93 patients included in this study are summarized in Table 1. The SOFA scores were significantly different between patients with and without bacteremia, and 20 patients with bacteremia (20 of 24; 83%) and 36 patients without bacteremia (36 of 69; 52%) were determined according to the criteria of definite sepsis. The platelet count was significantly lower in patients with bacteremia. Whilst IL-6, PCT, and presepsin levels were significantly higher in patients with bacteremia than in those without bacteremia.

**Table 1:** Clinical features and laboratory data of patients in the study.

	Total (*n* = 93)	Bacteremia (*n* = 24)	Non-bacteremia (*n* = 69)	*p*-value
Age, years	70 [56–78]	68.5 [63–81]	70 [52–77]	0.062
Male / Female	58/35	14/10	44/25	0.636
Underlying disease				
Diabetes mellitus	26 (28)	4 (17)	22 (32)	0.153
Malignancy	29 (31)	11 (46)	18 (26)	0.087
Severity				
SOFA score	2 [1–4]	4 [2–7]	2 [0–3]	0.002
Sepsis (≥2 SOFA score)	56 (60)	20 (83)	36 (52)	0.007
Laboratory data				
White blood cell (×10^3^/μL)	10.6 [7–14]	10.5 [6–13]	10.6 [8–14]	0.235
Neutrophils (×10^3^/μL)	8.8 [6–12]	9.5 [4.8–12]	8.7 [6–12]	0.410
Platelets (×10^4^/μL)	20.6 [15–28]	17.3 [13–22]	22.6 [16–29]	0.005
CRP (mg/dL)	7.1 [1.6–15]	8.4 [1.3–13]	6.7 [1.6–15]	0.268
Procalcitonin (ng/mL)	0.4 [0.1–1.6]	1.2 [0.3–21]	0.24 [1.0–1.7]	0.012
IL-6 (pg/mL)	204 [39–1,122]	1,122 [504–19,200]	116 [29–387]	0.012
Presepsin (pg/mL)	564 [366–1,200]	1,095 [550–1,590]	534 [338–867]	0.009
30-days mortality	0 (0)	0 (0)	0 (0)	—

Continuous variables are reported as median (interquartile range). Categorical variables are reported as number (percent). SOFA, sequential organ failure assessment; WBC, white blood cell; CRP, C-reactive protein; PCT, procalcitonin; IL-6, interleukin-6.

### Microbiological findings

The results of bacterial load and causative bacteria identified from blood culture are summarized in Table 2. Urinary tract and hepatobiliary tract/pancreatic infections were the most dominant organ-specific infections in patients with bacteremia, with a frequency of 50%. Bacterial load in the blood was detected by PCR in 16 patients (67%) with bacteremia and in 9 patients (13%) without bacteremia. Quantitative analysis showed that the bacterial load in the blood was significantly higher in patients with bacteremia than in those without bacteremia (*p* = 0.011). The most frequent causative bacteria for bacteremia were *Enterobacteriaceae* (15 cases; 63%), followed by *Staphylococcus* spp. (3 cases; 13%).

**Table 2:** Results of blood culture and Tm mapping in this study.

	Bacteremia (*n* = 24)	Non-bacteremia (*n* = 69)	*p*-value
Infected organ			
Lower respiratory tract	2 (8)	26 (38)	0.007
Urinary tract	7 (29)	13 (19)	0.289
Enteral/intra-peritoneal	3 (13)	8 (12)	0.906
Hepatobilliary/ pancreatic	5 (21)	4 (6)	0.032
Necrotizing fasciitis/ bone	2 (8)	6 (8)	0.956
Others	5 (21)	9 (13)	—
Focus unknown (bacteremia)	0 (0)	3 (4)	0.566
Blood bacterial load determined by PCR			
Positive (above detection limit)	16 (67%)	9 (13%)	<0.001
Copy/mL	525 [0–7,150]	0 [0–0]	0.011
Causative bacteria (cultured from blood)			
*Eschelia coli* + *Klebsiella* spp.	15 (63)	—	—
*Staphylococcus aureus*	3 (13)	—	—
*Pseudomonas aeruginosa*	1 (4)	—	—
Others	5 (21)	—	—

Continuous variables are reported as median (interquartile range). Categorical variables are reported as number (percent).

### Association of cell population data and bacteremia

**Figure 2:**
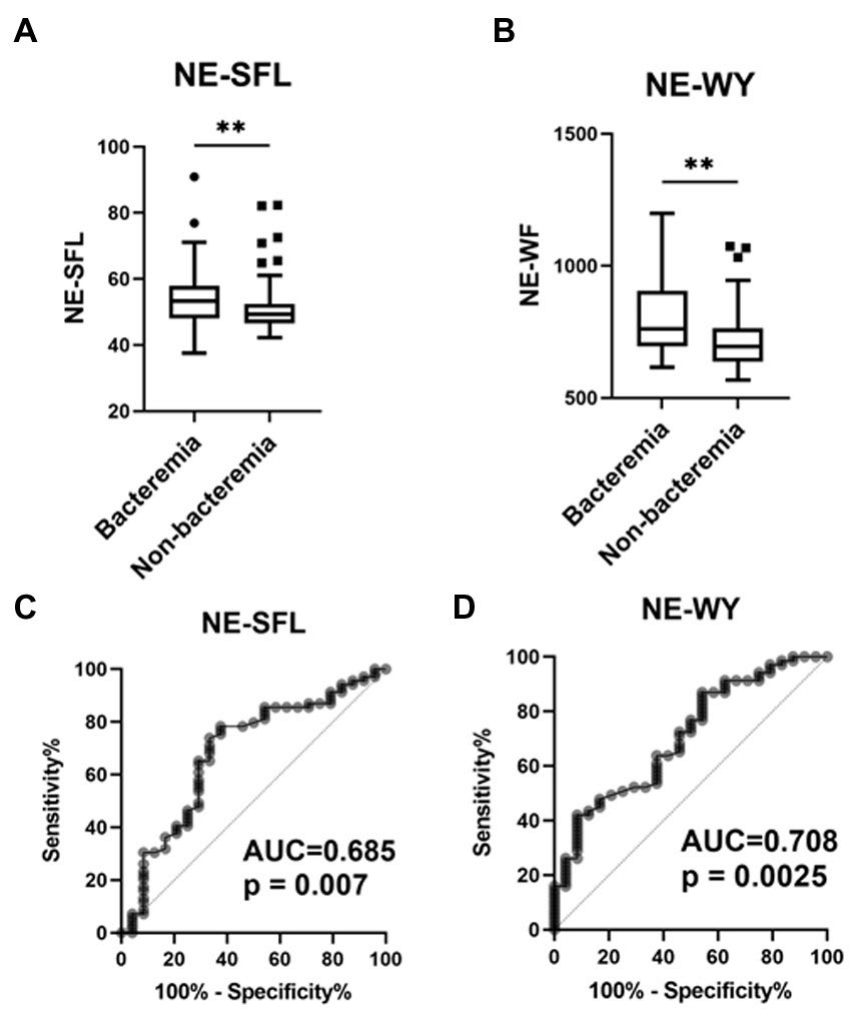
Value of the neutrophil parameters (NE-SFL and NE-WY) in patients who developed bacterial infection with or without bacteremia. NE-SFL **(A)** and NE-WY **(B)** in the patients with or without bacteremia. Data are presented as Tukey box-plots and individual values. ^**^p < 0.005. ROC, AUCs of NE-SFL **(C)** and NE-WY **(D)** for detecting bacteremia.

Among the cell population data measured using Sysmex’s automated hematology analyzer, NE-SFL and NE-WY were significantly higher in patients with bacteremia than in those without bacteremia (Figures 2A,B). There were no significant differences in CPD of lymphocytes and monocytes between patients with and without bacteremia (Supplementary Table S2).

Furthermore, ROC curves assessing these biomarkers for the diagnosis of bacteremia in acute infections were constructed and analyzed. The AUC of NE-SFL was 0.685 (sensitivity, 66.7%; specificity, 74.3%; cutoff value, 52.2), and that of NE-WY was 0.708 (sensitivity, 62.5%; specificity, 62.2%; cutoff value, 734) (Figures 2C,D). Among the sepsis biomarkers, IL-6 had the highest AUC (0.778), followed by PCT (0.744), presepsin (0.685), and CRP 0.528 (Supplementary Figure S1).

In an additional ROC curve analysis assessing the diagnostic value of these biomarkers for bacteremia in patients with definite sepsis (those with ≥2 SOFA score), the AUC of NE-SFL decreased to 0.632 (*p* = 0.190), while that of NE-WY increased to 0.744 (*p* = 0.005) (Supplementary Table S3).

**Figure 3:**
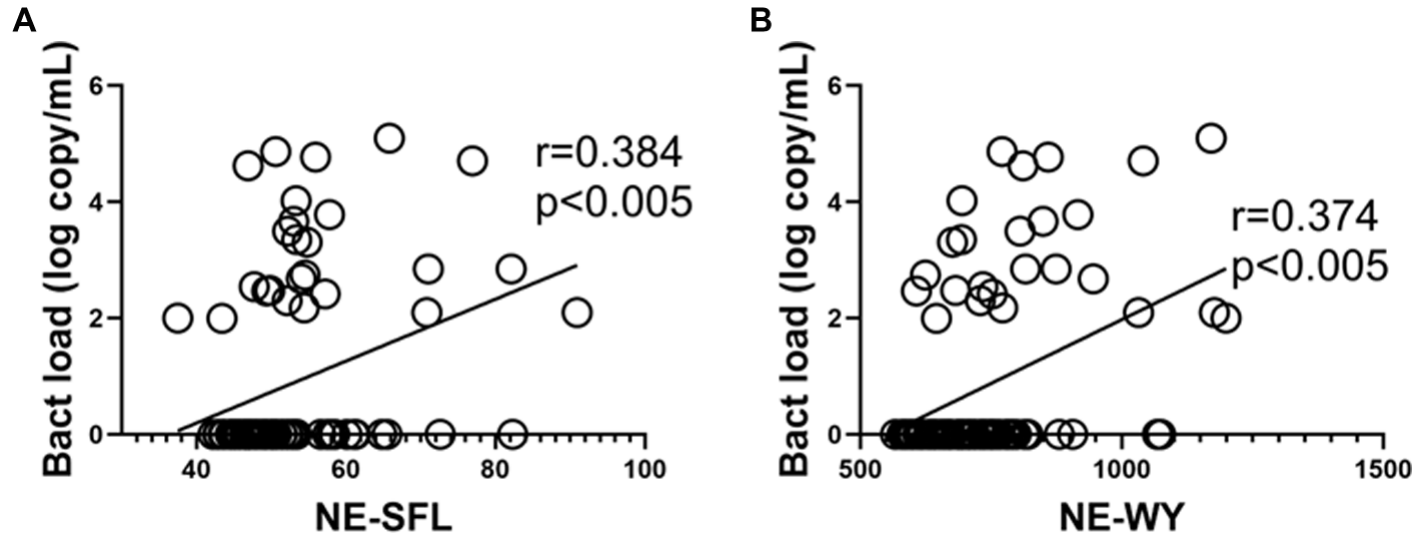
Correlations between the neutrophil parameters and bacterial load (determined by PCR) in patients who developed bacterial infection with or without bacteremia. Correlation between the bacterial load in blood and NE-SFL **(A)**/NE-WY **(B)**. Spearman correlation test was used, and Spearman correlation coefficient is shown. Corresponding logarithmic trendlines are shown.

As shown in Figures 3A,B, a similar positive correlation of NE-SFL and NE-WY with bacterial load was determined by PCR (*r* = 0.384 and 0.374, *p* < 0.005, respectively).

### Correlations among immunoinflammatory biomarker levels

**Figure 4:**
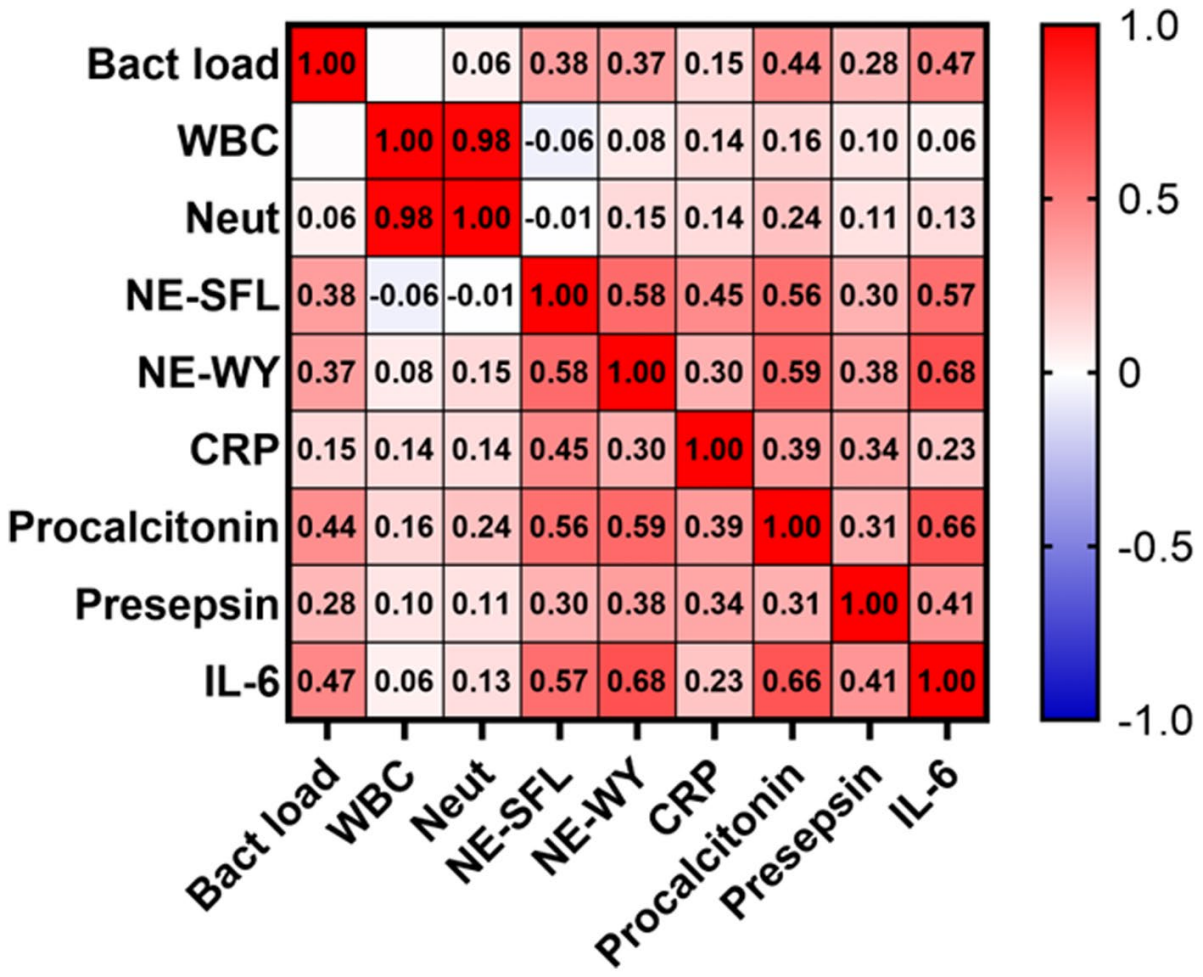
Correlation matrix of sepsis biomarkers and blood bacterial load in patients who developed acute bacterial infection. Results are presented as a correlation matrix. Spearman correlation coefficients are plotted. Cells were colored according to the strength and trend of correlations (shades of red = positive, shades of blue = negative correlations). ^*^p < 0.05. ^**^p < 0.001.

Among the tested sepsis biomarkers, IL-6 levels were most significantly correlated with NE-SFL (*r* = 0.57; *p* < 0.001) and NE-WY (*r* = 0.68; *p* < 0.001), followed by PCT (*r* = 0.56 and *r* = 0.59) and presepsin (*r* = 0.30 and *r* = 0.38, respectively) (Figure 4). However, leukocyte and neutrophil counts did not significantly correlate with NE-SLF or NE-WY.

In an additional analysis assessing the correlation between these biomarkers in definite sepsis patients, the correlation between NE-SFL/NE-WY and other parameters was similar to that in patients with acute infection (Supplementary Figure S2).

## Discussion

This study demonstrated the significant diagnostic value of NE-SFL and NE-WY for detection of bacteremia in patients with acute infection. The definite correlation between blood bacterial load and NE-SFL/NE-WY, determined by PCR, demonstrate that these parameters are affected by initial bacterial invasion into the blood. Among the sepsis biomarkers, NE-SFL/NE-WY were strongly associated with PCT and IL-6, but not with presepsin or CRP. To the best of our knowledge, this study is the first to document an association between CPD and sepsis biomarkers, including IL-6 and presepsin in acute bacterial infection.

In this study, we assessed the diagnostic value of NE-SFL and NE-WY in patients who developed acute bacterial infections, with or without culture-proven bacteremia. The ROC curve for bacteremia in bacterial infection analyzed using NE-SFL and NE-WY revealed a relatively high AUC of 0.685 and 0.708, respectively. Together with the definite correlation between NE-SFL/NE-WY and bacterial load in the blood, we suggest that NE-SFL and NE-WY both have predictive value to detect bacteremia among patients with acute infection. Moreover, NE-WY showed a relatively strong correlation with PCT and IL-6 in patients with definite sepsis compared to NE-SFL, as shown in Supplementary Table S3. These differences may be partly due to the nature of NE-WY, which potentially reflects infection-related cell death rather than cell proliferation.

NE-SFL and NE-WY are postulated to reflect the immaturity or activation of neutrophils because high fluorescence intensity indicates a high RNA/DNA ratio in immature cells. NE-SFL reflects the increase in proportion of the amount of cellular DNA and RNA, while NE-WY reflects the degree of heterogeneity of the neutrophil population. In the early phase of bacteremia, the mobilization of juvenile leukocytes with high nucleic acid content increases in the peripheral blood, resulting in a high NE-SFL (16). Simultaneously, neutrophils undergo cell death through several mechanisms after responding to bacteria, including apoptosis, necrosis, and NETosis (17–19), which is reflected in the variety of nucleic acids, possibly resulting in high NE-WY values. Since severe bacterial infection induces cell death in addition to the proliferation of juvenile neutrophils, a stronger association with severity or sepsis markers might be observed with NE-WY than with NE-SFL.

In this study, we determined the quantitative bacterial load of patients using the PCR method (15), which is part of the ‘melting temperature mapping (Tm mapping)’ method (15). Tm mapping is a novel molecular genetic method for identifying a broad range of pathogenic bacteria using a real-time PCR-based system. In attempt to detect a wide range of bacteria, the Tm mapping method is designed with seven bacterial universal primer sets targeting bacterial conserved regions in 16S ribosomal RNA gene in a nested PCR assay to detect and identify bacterial isolates with high sensitivity and specificity; the Tm mapping method was able to detect 95.6% of culture-proven bacteremia in the analysis using 200 blood samples (15). Using this PCR method, we confirmed that the value of sepsis biomarkers, including NE-WY/NE-SFL, was significantly correlated with bacterial load in patients with acute bacterial infection. Interestingly, NE-WY strongly correlated with bacterial load in the blood of patients with definite sepsis, which might be induced by an increase in cell death or phagocytosis of bacteria.

Among different biomarkers which have been proposed to predict sepsis or bacteremia, the main attributes of successful and effective biomarkers are high sensitivity, specificity, possibility of bed-side monitoring, and financial accessibility (20). To date, the sepsis biomarkers that commercially available in Japan are CRP, PCT, presepsin and IL-6. Emerging evidence has suggested several other biomarkers as novel diagnostic tools in acute bacterial infections, such as lipopolysaccharide-binding protein (21), interleukins and cytokines other than IL-6 (e.g., IL-8, IL-10, TRAIL) (22, 23), surface markers of circulating leukocytes such as Cluster of Differentiation 64 (CD64) (24), and precursor of hormones such as mid-regional fragment pro-adrenomedullin (25). To establish more accurate and efficient diagnostic procedures, the combination of biomarkers and diagnostic methods has been investigated as a novel diagnostic approach in bacterial infection (26, 27).

In this study, IL-6 and PCT showed the highest predictive value for the presence of bacteria, whereas presepsin and CRP showed a relatively lower predictive value, than NE-WY/NE-SFL. Of note, the sepsis biomarkers IL-6, PCT, presepsin and CRP are released from various other non-neutrophil immune cells (22, 28). The significant association between NE-WY/NE-SFL, which reflect the immune response in neutrophils, and IL-6/PCT, suggests potential benefits in the use of a combination of these biomarkers in predicting severe bacterial infections.

Presepsin, the soluble fraction of cluster of differentiation 14 (CD14), is a sepsis biomarker that is released into circulation when monocytes are activated after binding with lipopolysaccharides (LPS) and LPS-binding proteins (29, 30). Recently, Park et al. (31) reported that presepsin showed a higher AUC than PCT (0.720 and 0.593; *p* = 0.002) for the prediction of 28-day mortality in 757 patients with bacterial infection, whereas the AUC of presepsin for detecting culture-proven bacteremia was lower than that of PCT (0.685 and 0.791; *p* < 0.001). As NE-SFL/NE-WY showed a higher AUC than presepsin, we consider presepsin to predict mortality rather than the presence of bacteremia in sepsis patients. The lack of fatal sepsis cases in this study may have also affected the diagnostic value of presepsin.

Based on these findings, we suggest the potential synergistic benefit and interaction between NE-WY/NE-SFL and conventional sepsis biomarkers. As NE-WY/NE-SFL determination incurs no additional costs other than routine examination by the automated differential, these CPD parameters have strong potential to be routine sepsis biomarkers that predict bacteremia or severe infection in acute bacterial infections.

This study had several limitations. First, the single-center observational study design may have resulted in selection bias. Second, the relatively small sample size, particularly regarding patients who developed culture-proven bacteremia, may limit the reproducibility of the results. However, because this study focused on the association between CPD, sepsis biomarkers, and bacterial load, we believe that these limitations did not have a major effect on our conclusions.

## Conclusion

In this study, we demonstrated that NE-SFL and NE-WY measured using the Sysmex XN-2000 have a high diagnostic efficacy for prediction of bacteremia in acute bacterial infections. We also found that NE-SFL and NE-WY are strongly associated with the blood bacterial load, determined by PCR. In addition to the diagnostic value and substantial financial accessibility, the significant association with conventional sepsis biomarkers suggest potential benefits of NE-WY/NE-SFL in routine use in predicting severe bacterial infections. To further advance the early detection and understanding of bacteremia in acute bacterial infections, more investigation into the diagnostic value of CPD, particularly with NE-WY and NE-SFL is warranted.

## Data availability statement

The original contributions presented in the study are included in the article/Supplementary material, further inquiries can be directed to the corresponding author.

## Ethics statement

The studies involving human participants were reviewed and approved by Toyama University Hospital Ethics Committee (29–152). The patients/participants provided their written informed consent to participate in this study.

## Author contributions

YM and HN designed and interpreted the clinical data and experimental findings. YM, KN, and HN prepared the manuscript. YM with the assistance of TU, AM, YH, NK, and MK collected the clinical data and blood. YM and HN contributed to the analysis of the experimental and microbiological findings. YM and KN confirmed the accuracy of the statistical analysis. YM, HN, TU, AM, YH, NK, MK, YI, KN, YY, and IK contributed to the discussions throughout the work. All authors contributed to the article and approved the submitted version.

## Funding

HN received research funding on a contract basis and joint research funding from Sysmex Corporation.

## Conflict of interest

NK and YI were employed by the company Sysmex Corporation. Author MK was employed by the company Subsidiary of Sysmex Corporation. The authors declare that this study received funding from Sysmex Corporation. The funder had the following involvement in the study: study design, analysis, interpretation of data.

The remaining authors declare that the research was conducted in the absence of any commercial or financial relationships that could be construed as a potential conflict of interest.

## Publisher’s note

All claims expressed in this article are solely those of the authors and do not necessarily represent those of their affiliated organizations, or those of the publisher, the editors and the reviewers. Any product that may be evaluated in this article, or claim that may be made by its manufacturer, is not guaranteed or endorsed by the publisher.

## Supplementary material

The Supplementary material for this article can be found online at: https://www.frontiersin.org/articles/10.3389/fmed.2023.1156889/full#supplementary-material
