# Supplementary material for: Predictive value of cell population data with Sysmex XN-series hematology analyzer for culture-proven bacteremia
Source: Front Med (Lausanne). 2023 Jun 1;10:1156889. doi: 10.3389/fmed.2023.1156889 (PMC10267328; doi:10.3389/fmed.2023.1156889)
Supplement: Supplementary file 1 [file Data_Sheet_1.docx]

Online Depository

Supplementary Figure and Table

**Predictive value of cell population data with Sysmex XN-series hematology analyzer for culture-proven bacteremia**

**Table 1**. The morphological and functional characteristics of the whole panel of CPD measured by WDF channel of Sysmex XN-2000.

| **Leukocyte parameters** | **Analysis method of leukocyte cluster** | **Reflecting component** |
| --- | --- | --- |
| SSC | side scattered light | internal cell structure and granularity |
| SFL | side fluorescent light | mainly DNA/RNA content |
| FSC | forward scattered light | cell size |
| WX | side scattered light distribution width index | variation in internal cell structure and granularity |
| WY | fluorescent light distribution width index | variation in mainly DNA/RNA content |
| WZ | forward scattered light distribution width index | variation in cell size |

**Table 2** Value of the cell population data in patients who developed bacterial infection with or without bacteremia.

| **Leukocyte parameters** | **Bacteremia**  (n=24) | **Non-bacteremia**  (n=69) | **Healthy control**  **(**n=37) |
| --- | --- | --- | --- |
| NE-SSC | 155.4 (153–160) | 155.1 (151–158) | 152.4 (151–155) |
| NE-SFL | 53.5 (48–58) | 49.3 (47–53) | 46.8 (45–49) |
| NE-FSC | 90.6 (86–95) | 87.0 (84–92) | 88.5 (87–92) |
| NE-WX | 304.5 (289–319) | 308.0 (301–321) | 292 (286–305) |
| NE-WY | 761.5 (694–906) | 694.0 (636–763) | 600 (587–621) |
| NE-WZ | 766.5 (691–875) | 736.0 (678–811) | 754 (699–799) |
| Ly-SSC | 82.1 (81–83) | 81.8 (81–84) | 80.9 (80–82) |
| Ly-SFL | 67.2 (63–70) | 67.8 (65–71) | 68.4 (66–70) |
| Ly-FSC | 56.6 (54–60) | 56.4 (54–59) | 56.1 (55–58) |
| Ly-WX | 441.5 (412–492) | 465.0 (431–520) | 496.0 (468–530) |
| Ly-WY | 879.0 (671–961) | 857.0 (773–978) | 861.0 (815–890) |
| Ly-WZ | 651.5 (562–715) | 585.0 (541–629) | 636.0 (562–686) |
| Mo-SSC | 124.1 (121–127) | 121.9 (120–124) | 120.9 (120–122) |
| Mo-SFL | 111.2 (103–124) | 112.2 (108–119) | 111.2 (109–115) |
| Mo-FSC | 66.0 (64–69) | 66.6 (64–69) | 68.2 (67–70) |
| Mo-WX | 261.5 (237–311) | 267.0 (238–293) | 244.0 (226–266) |
| Mo-WY | 688.5 (292–783) | 719.0 (664–795) | 656.0 (597–714) |
| Mo-WZ | 704.5 (535–813) | 668.0 (603–747) | 690.0 (565–802) |

Values are presented as median (IQR).


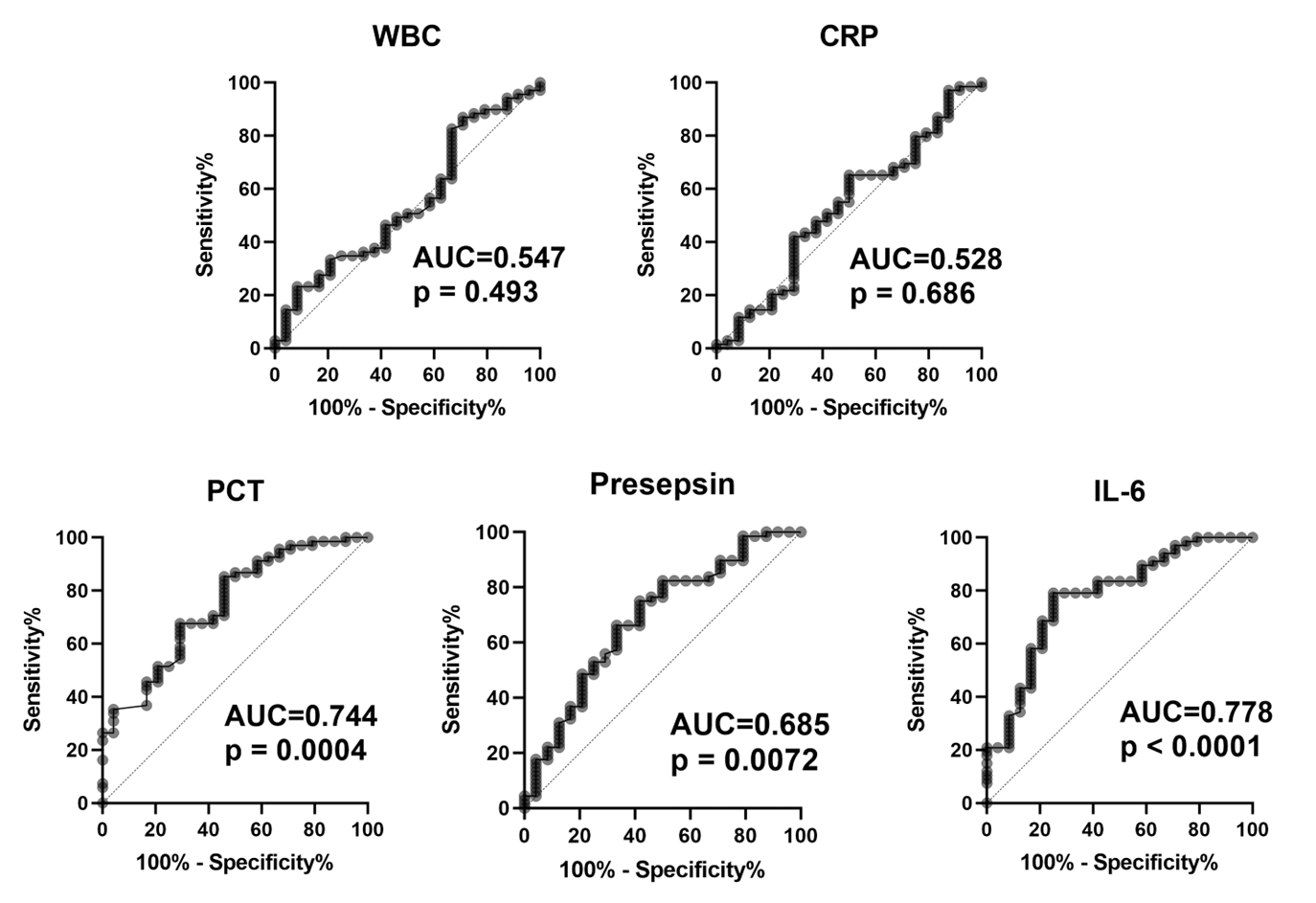


**Supple Fig 1**

Receiver operating curve (ROC) assessment of sepsis biomarkers for the diagnosis of bacteremia in acute infections.

**Supple Table 3.** Receiver operating curve (ROC) assessing sepsis biomarkers and SOFA score for the diagnosis of bacteremia in patients with definite sepsis, SOFA score ≥2.

| **Biomarker** | **AUC** | **95%CI** | ***p*　value** |
| --- | --- | --- | --- |
| Bact load | 0.744 | 0.57–0.92 | 0.007 |
| SOFA | 0.994 | 0.98–1.00 | <0.005 |
| WBC | 0.674 | 0.47–0.88 | 0.010 |
| Neut | 0.680 | 0.48–0.88 | 0.079 |
| NE-SFL | 0.632 | 0.43–0.83 | 0.190 |
| NE-WY | 0.744 | 0.58–0.91 | 0.005 |
| CRP | 0.605 | 0.43–0.78 | 0.250 |
| Procalcitonin | 0.812 | 0.68–0.94 | <0.005 |
| Presepsin | 0.694 | 0.54–0.85 | 0.017 |
| IL-6 | 0.798 | 0.67–0.92 | <0.005 |


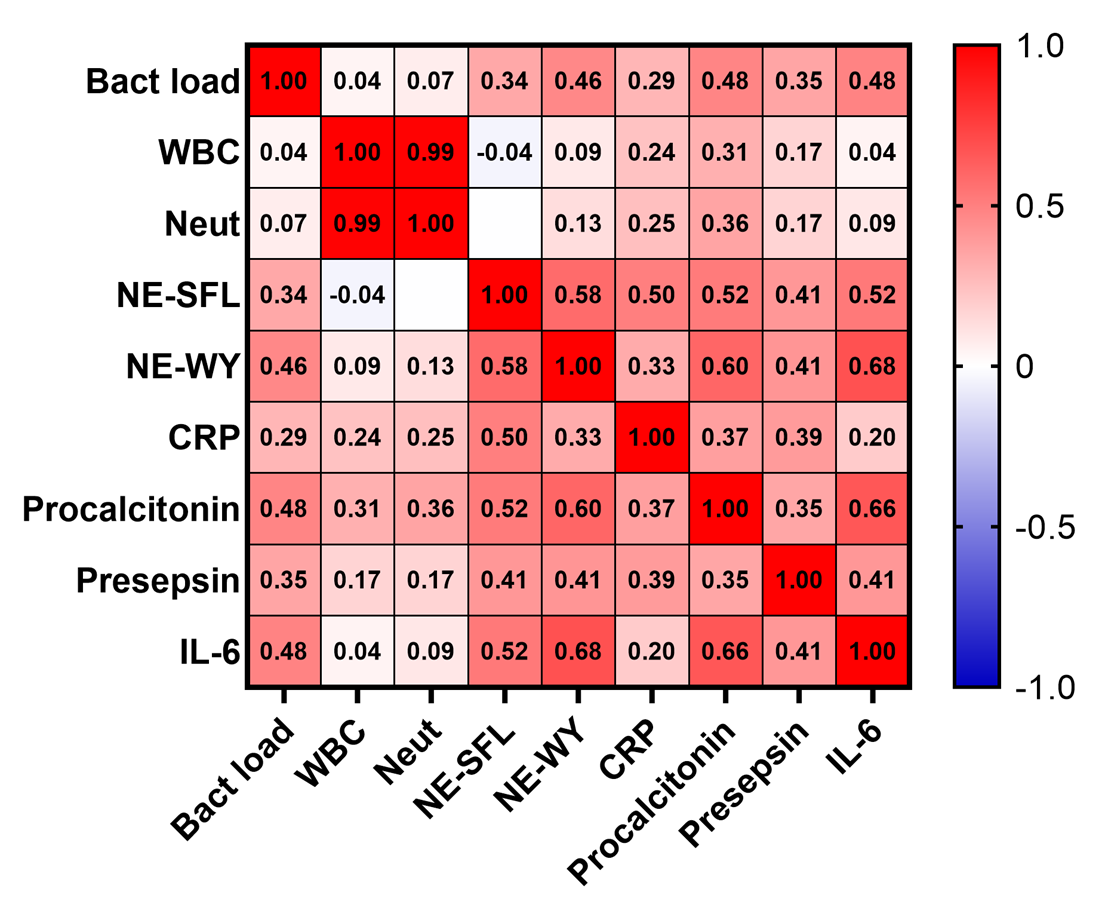


**Supple Figure 2**

Correlation matrix of sepsis biomarkers, genetically determined blood bacterial load, and severity score in patients who developed definite sepsis. The results are presented as a correlation matrix. The Spearman’s correlation coefficients were plotted. Cells are colored according to the strength and trend of correlations (shades of red = positive, shades of blue = negative correlations). ^＊^: *p*<0.05. ^＊＊^: *p*<0.001.
